# Coherent Light Photo-modification, Mass Transport Effect, and Surface Relief Formation in As_x_S_100-x_ Nanolayers: Absorption Edge, XPS, and Raman Spectroscopy Combined with Profilometry Study

**DOI:** 10.1186/s11671-017-1918-y

**Published:** 2017-02-27

**Authors:** O. Kondrat, R. Holomb, A. Csik, V. Takáts, M. Veres, V. Mitsa

**Affiliations:** 1 Uzhhorod National University, Pidhirna Str. 46, Uzhhorod, 88000 Ukraine; 2 Institute for Nuclear Research, Hungarian Academy of Sciences, Debrecen, H-4001 Hungary; 3 Wigner Research Centre for Physics, Hungarian Academy of Sciences, Budapest, 1121 Hungary

**Keywords:** As-S nanolayers, Photoinduced changes, XPS, Raman spectroscopy, Core level, Valence band, Mass transport, Chalcogenide thin films

## Abstract

As_*x*_S_100-*x*_ (*x* = 40, 45, 50) thin films top surface nanolayers affected by green (532 nm) diode laser illumination have been studied by high-resolution X-ray photoelectron spectroscopy, Raman spectroscopy, optical spectroscopy, and surface profilometry. It is shown that the composition of obtained films depends not only on the composition of the source material but as well on the composition of the vapor during the evaporation process. Near-bandgap laser light decreases both As–As and S–S homopolar bonds in films, obtained from thermal evaporation of the As_40_S_60_ and As_50_S_50_ glasses. Although As_45_S_55_ composition demonstrates increasing of As–As bonds despite to the partial disappearance of S–S bonds, for explanation of this phenomenon Raman investigations has also been performed. It is shown that As_4_S_3_ structural units (s.u.) responsible for the observed effect. Laser light induced surface topology of the As_45_S_55_ film has been recorded by 2D profilometer.

## Background

The research of chalcogenide glassy (ChG) materials formed a general understanding of electronic phenomena in disordered structures [1, 2]. The numerous investigations of their fundamental physical and chemical properties have been already performed [3–6]. Unique structural, electronic, and optical properties determined their various applications. The high infrared (IR) transparency of fibers on the basis of the ChG allows transmitting high-power IR light. The large refractive indices and third-order optical nonlinearities of the chalcogenide glasses make them the best candidates for the photonic devices for ultrafast all-optical switching and data processing [7]. Various applications have been proposed on the basis of the light sensitivity of non-crystalline chalcogenides, especially in amorphous thin film form [8–10]. Thus, photosensitivity is the main feature of chalcogenide glasses for phase-change memory, direct waveguides, and grating patterning.

The high-quality optical elements are required for the development of all-optical signal processing systems. Possibility of high-level integration of these elements in optical chips implies improved fabrication technology in order to achieve low optical losses at the near surface layers and the high level of laser damage threshold at femtosecond laser pulses. Also the large IR transparency or high optical nonlinearity of amorphous As–S binary systems make them a prospective optical media for the future ultrafast photonic systems. Our previous Raman studies of non-crystalline As–S binary system reveal the differences between the structures of As_2_S_3_ films and bulk glass at nano-scale dimension [11]. The analysis shows that it is caused mainly by the phase separation, i.e., contribution of As_4_S_4_ cage-like molecules in the vapor during As_2_S_3_ thermal evaporation. In the further studies of the structure of amorphous As_2_S_3_ glasses and films using photon-energy-dependent Raman spectroscopy, the effect of laser-induced transformation of As_4_S_4_ molecules was observed [12]. Therefore, the As_4_S_4_ molecules can be classified as light absorption centers in As_2_S_3_ structure and leading to increasing the optical losses of an optical media.

The structure and properties of As_45_S_55_ glassy material and thin film were investigated earlier [13, 14]. Using macro FT-Raman spectroscopy, energy-dependent micro-Raman spectroscopy and first principle calculations established that the light-induced structural transformations in As_45_S_55_ glass take place mainly from alterations of As_4_S_4_ molecules in glass network. An impact of near-bandgap laser illumination transforms α(β)-As_4_S_4_ molecules to pararealgar-like p-As_4_S_4_ [13]. The extended X-Ray absorption fine structure (EXAFS) study of photoinduced structural changes in amorphous As_*x*_S_1-*x*_ thin films showed that effect of the near-bandgap light illumination to the evaporated a-As_42_S_58_ and a-As_45_S_55_ films results in more disordered state and photostructural transformations are related to changes in the amorphous As-S network [14].

Raman spectroscopy of As-rich As_50_S_50_ thin films revealed pararealgar structure of χ-As_4_S_4_, β-As_4_S_4_ molecules, clusters of amorphous arsenic, S_2_As-AsS_2_ and As_4_S_5_ structural units (s.u.), some part of α-As_4_S_4_, As_4_S_3_ molecules, and AsS_3_ pyramids [15].

The aim of the present work is a complex structural investigation of thin film surface nanolayers prepared from As_40_S_60_, As_45_S_55_, and As_50_S_50_ chalcogenide glasses using X-ray photoelectron (XPS) and Raman spectroscopy, near-bandgap laser light’s influence on structural and compositional changes, and their electronic structure. In addition, the changes of surface morphology induced by laser light illumination were investigated using surface profilometry method.

## Methods 

High-quality optical glasses were used as the source materials for sample deposition in order to avoid the contamination in the volume of the films. The bulk As_*x*_S_100-*x*_ (*x* = 40, 45, 50) samples were prepared by conventional melt-quenching route in evacuated quartz ampoules from a mixture of high purity 99.999% As and S precursors. Nanolayers were prepared by thermal vacuum evaporation of appropriate bulk glass powders onto silicon and glass substrates. The thicknesses of obtained films were ~0.7 μm. Green diode laser operating at λ = 532 nm wavelength (photon energy of ~2.4 eV) with power *p* = 25 mW was used to investigate the influence of the near-bandgap light irradiation on the samples of As_40_S_60_, As_45_S_55_, and As_50_S_50_ (E_g_ ~2.4 eV) nanolayers. Optical irradiation was carried out with 280 mW/cm^2^ intensity at ambient conditions. Laser intensity was chosen based on our previous studies of As–S glasses by means of Raman spectroscopy, mentioned above. To determine exposition of the laser illumination following experiments was done. The absorption edge of the As_*x*_S_100-*x*_ (*x* = 40, 45, 50) thin films and its shift under in situ illumination by green laser light was investigated using millisecond CCD spectrometer ThorLabs CCS200. Sample illumination with in situ optical spectra recording were done until saturation of the shift of the absorption edge.

Photoemission experiments were conducted by using Al k-α anode (E = 1486 eV) as a source of X-ray. Spectra were recorded using hemispherical energy analyzer series Phoibos 100. An As 3d and S 2p core levels and valence bands (VB) were measured at normal emission geometry. Apart from this, the C 1s and O 1s core level spectra were recorded in order to normalize the positions of all spectra to a position of the graphitic peak (at 284.5 eV, [16]). C 1s core level spectra were fitted by C–C and C–O components only, and this agrees with the O–C components founded in O 1s core level spectra. Due to that C 1s and O 1s core levels would not be included in the further consideration. The CASA XPS program was used to fit core level spectra. For core level fitting, the Voigt profile components were used and Shirley background was subtracted.

Raman spectra were measured with using Renishaw system 1000 Raman spectrometer, equipped with a CCD detector. The diode laser operating at 785 nm was used as the excitation source. The measurements were made in micro-Raman configuration with using back-scattering geometry. In order to avoid stimulated by this laser photoinduced changes in the structure of the samples, the output power of the excitation source was limited by optical filters [17].

## Results

### Absorption Edges of As_*x*_S_100-*x*_ Films Under External Influence

**Fig. 1:**
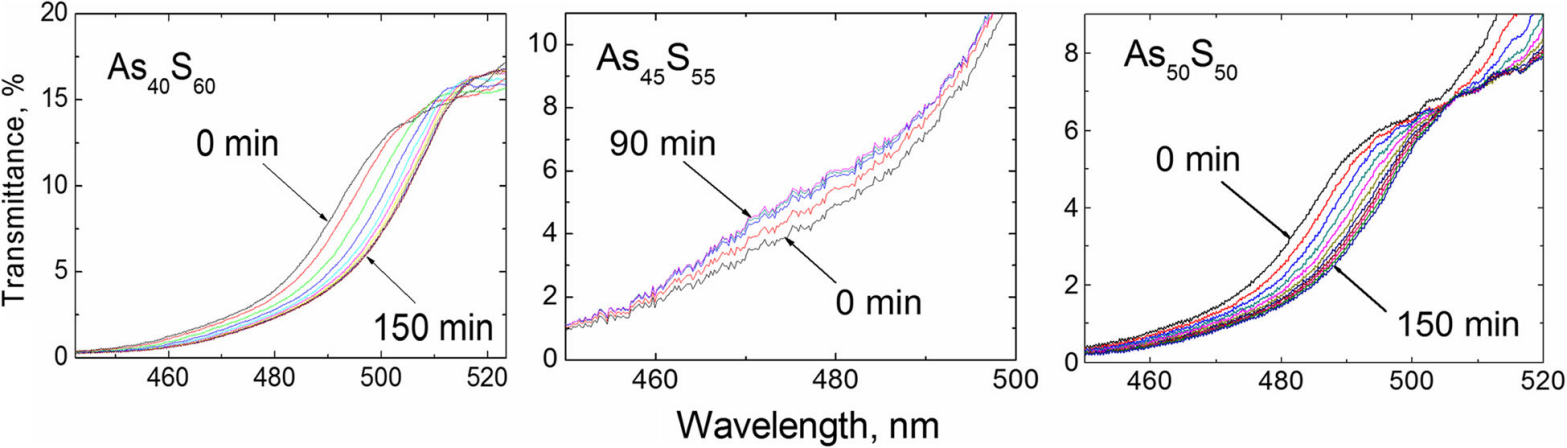
A shift of absorption edge of As_40_S_60_, As_45_S_55_, and As_50_S_50_ thin films under green (λ = 532 nm) laser light illumination

Optical spectra of absorption edges of the As_40_S_60_, As_45_S_55_, and As_50_S_50_ films are shown in Fig. 1. As can be seen, the absorption edge of As_40_S_60_ sample shifts towards longer wavelengths when film exposed to green laser light illumination (λ = 532 nm) with photon energy of ~2.4 eV, which is very close to E_g_ of As_40_S_60_ material (Fig. 1, left). Typical red shift of the absorption edge of As–S films during the near bandgap illumination was observed earlier [5, 18]. After 45 min of laser illumination of As_40_S_60_ film, the shift becomes less and at exposure time of ~150 min, the changes almost disappeared.

A similar phenomenon was observed for the absorption edge of illuminated As_50_S_50_ film (Fig. 1, right). However, the results of the same investigation of As_45_S_55_ film demonstrate the opposite effect. For this composition, the blue shift of the absorption edge is observed under the green laser illumination and 90 min was enough to reach the saturation. The structural interpretation of this phenomenon will be provided in the next paragraph using the XPS analysis and Raman spectroscopy data.

The determined particular exposure times sufficient to saturate the changes of the absorption edges of As_40_S_60_ (t_exp._ = 150 min), As_45_S_55_ (t_exp._ = 90 min), and As_50_S_50_ (t_exp._ = 150 min) films were used for further experiments.

### XPS Spectroscopy and Valence Band Spectra of As_*x*_S_100-*x*_ (*x* = 40, 45, 50) Film Surfaces

**Fig. 2:**
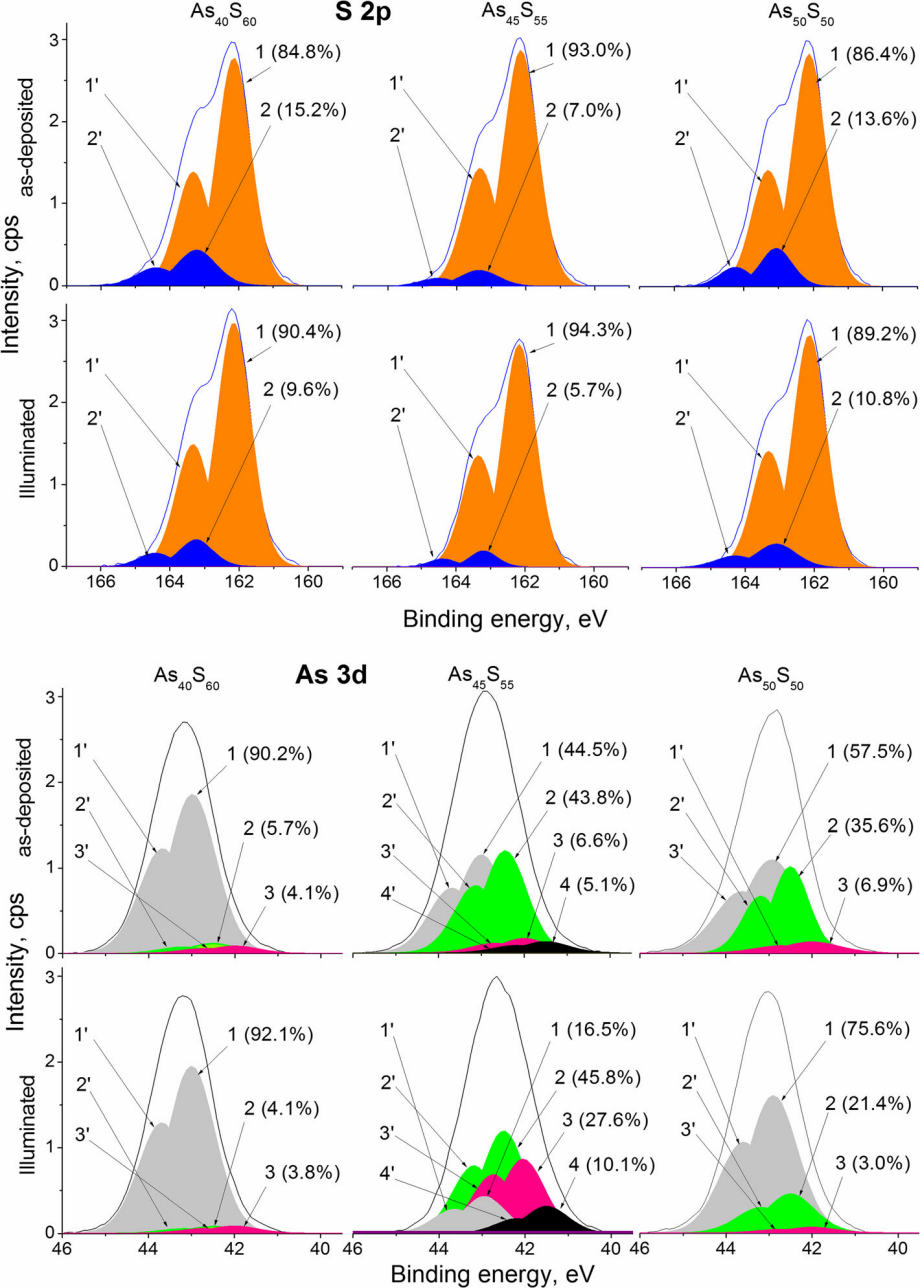
S 2p and As 3d fitted core level spectra of as-deposited (*top*) and illuminated by green (λ = 532 nm) laser (*bottom*) As_40_S_60_, As_45_S_55_, and As_50_S_50_ thin films top nanolayers. For S 2p: *1*, *2* denote 2p_3/2_ and *1´*, *2´* denote 2p_1/2_ peaks of S–As_2_ (1, 1’) and S-SAs (2, 2’) components; for As 3d: *1*, *2*, *3*, *4* denote 3d_5/2_ and *1´*, *2´*, *3´*, *4’* denote 3d_3/2_ peaks of As–S_3_ (1, 1’), As–S_2_As (2, 2’), As–SAs_2_ (3, 3’), and As–As_3_ (4, 4’) components

X-ray photoelectron spectroscopy can be a useful technique to investigate the surface (i.e*.* few topmost layers) of the materials at short-range order scale and to determine the structural units which form the investigated material. This method was successfully used to characterize the top surface nanolayer structure of the amorphous materials [19–21]. The results of XPS investigation of As_*x*_S_100-*x*_ (*x* = 40, 45, 50) thin film surface nanolayers are summarized in Fig. 2. As can be seen, all S 2p core level spectra can be fitted by two components. The energy position of component 1 (and their spin-orbit split 1’) allows assigning them to S–As_2_ s.u. [22, 23]. It is expected that this component is a characteristic s.u. in crystalline As_2_S_3_ and is a main component for both stoichiometric, As_40_S_60_, and As-rich As–S glasses and films. The component 2 (and 2’) can be assigned to S-rich S–SAs s.u., and it is in a good agreement with our earlier investigations and theoretical estimations [19]. The As 3d core level spectra of As_40_S_60_ nanolayers (both as-deposited and illuminated by green laser light) are fitted using three components: arsenic bonded to three sulfur atoms As–S_3_ (1, 1’), arsenic bonded to two sulfur, and one arsenic atoms As–S_2_As (2, 2’) and finally, arsenic bonded to one sulfur and two arsenic atoms As–SAs_2_ (3, 3’). These assignments are based on our previous study [19] and are in excellent agreement with the published data [16, 24]. It should be noted that for the best fit of the As 3d core level spectra of As_45_S_55_ nanolayers, the fourth component is needed. The position of this component allows to interpret it as arsenic bonded to three arsenic atoms [16]. All peak component parameters are listed in Table 1.

**Table 1:** Binding energies (BE, ±0.1 eV) and full width at half maximum (FWHM) (±0.05 eV) data of individual components determined from curve fitting of S 2p and As 3d XPS spectra of as-deposited and illuminated by green (λ = 532 nm) laser As_40_S_60_, As_45_S_55_, and As_50_S_50_ nanolayers

Core level/component	As_40_S_60_				As_45_S_55_				As_50_S_50_			
	As received		Illuminated		As received		Illuminated		As received		Illuminated	
	BE	FWHM	BE	FWHM	BE	FWHM	BE	FWHM	BE	FWHM	BE	FWHM
**S 2p:**												
S–As_2_	162.1	1.2	162.1	1.2	162.1	1.2	162.2	1.1	162.1	1.1	162.1	1.1
S–SAs	163.2	1.3	163.2	1.1	163.3	1.3	163.2	0.9	163.1	1.1	163.1	1.3
**As 3d:**												
As–S_3_	43.0	1.3	43.0	1.3	43.0	1.3	42.9	1.2	42.9	1.5	42.9	1.3
As–S_2_As	42.5	1.3	42.5	1.3	42.4	1.2	42.5	1.2	42.5	1.0	42.5	1.3
As–SAs_2_	42.0	1.3	42.0	1.3	42.0	1.3	42.1	1.2	42.0	1.5	42.0	1.3
As–As_3_	–	–	–	–	41.5	1.3	41.5	1.2	–	–	–	–

**Fig. 3:**
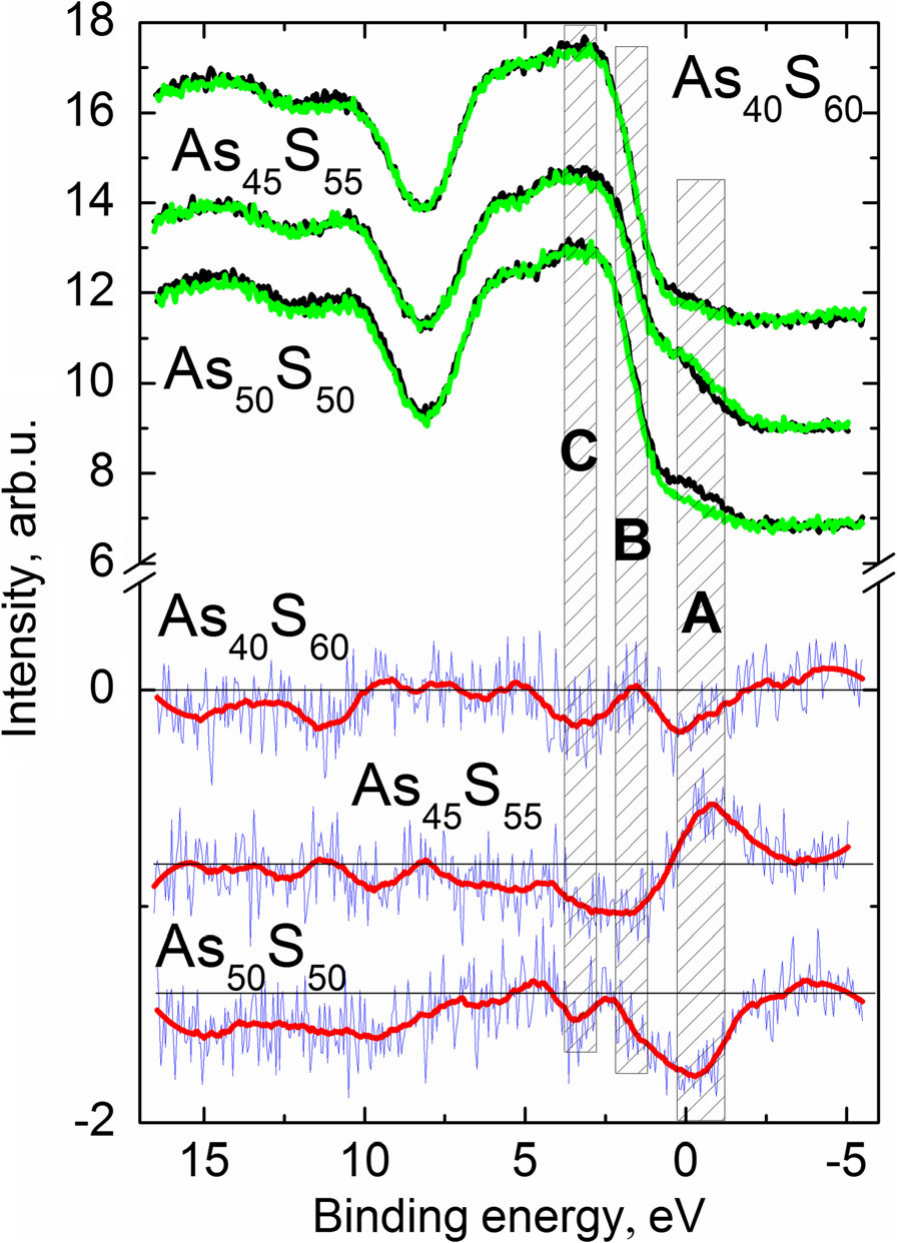
Valence band spectra of as-deposited (*black lines*) and illuminated by green (λ = 532 nm) laser light (*green lines*) As_40_S_60_, As_45_S_55_, and As_50_S_50_ thin film nanolayers (*top part*). Differential (illuminated minus as received) original (*blue spiked lines*) and smoothed (*red thick lines*) spectra (*bottom part*)

The valence band spectra of as-deposited and illuminated by laser As_*x*_S_100-*x*_ (*x* = 40, 45, 50) thin film surface nanolayers were also measured and shown in Fig. 2. The general view of these spectra for all As–S compositions is similar and correlates well with the valence band spectra of As_40_S_60_ films [25, 26]. For a better understanding of the structural changes caused by laser light illumination, differential VB spectra were constructed (Fig. 3, bottom part).

### Raman Spectroscopy of As_40_S_60_, As_45_S_55_, and As_50_S_50_ Glasses and Films

Results of XPS measurements give a possibility to analyze the structure of investigated materials at micro-level (short-range order) and to determine structural units which form the substance. For a better understanding of the nature of processes and stimulated structural changes, it is necessary to investigate the structure of the samples at the extended scale range (i.e., medium range order) in order to determine the macrostructure of materials. The cage-like molecules, rings, chain-like and bigger clusters in the structure of the As–S glasses and films can clearly be detected and identified from the Raman spectra [27]. Also, the photon energy-dependent micro-Raman spectroscopy can successfully be used for monitoring the photoinduced molecular transformations [7, 12]. Therefore, this technique was used for complex investigation of the structure and induced transformations in As_40_S_60_, As_45_S_55_, and As_50_S_50_ films.

**Fig. 4:**
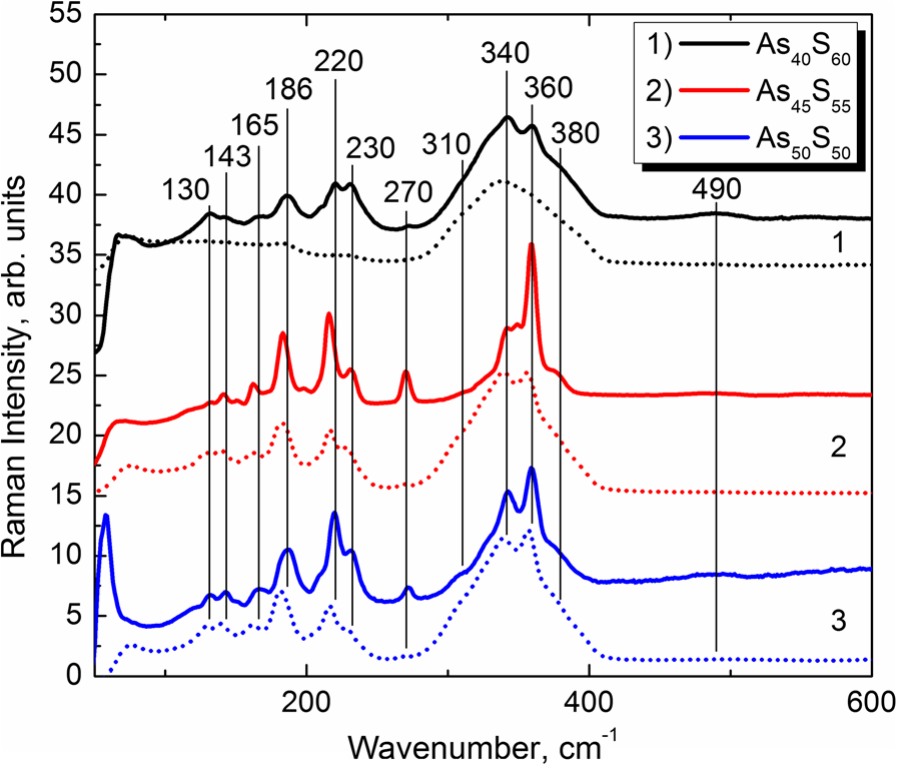
Raman spectra of As_40_S_60_ (*1*), As_45_S_55_ (*2*), and As_50_S_50_ (*3*) target glasses (*dotted line*) and corresponding thin films (*solid line*)

The Raman spectra of source As_40_S_60_, As_45_S_55_, and As_50_S_50_ glasses and corresponding As–S thin films are shown in Fig. 4. As can be seen, the Raman spectra of g-As_40_S_60_ demonstrate a broad band with the maximum at 340 cm^−1^ and shoulders at 310 and 380 cm^−1^. The main band centered at 340 cm^−1^ is a characteristic band of symmetric As–S vibrations in AsS_3_ pyramids. The shoulders at 310 and ~380 cm^−1^ are connected with the assymetric As–S vibrations in AsS_3_ pyramids and As–S–As vibrations of “water-like” molecule, respectively. The Raman band at 130 cm^−1^ can be connected with deformational vibrations of –As–S–As– and –S–As–S– structures. In addition to these bands, the very weak features at 143, 165, 186, 220, 230, and 360 cm^−1^, connected with homopolar As–As bonds and realgar As_4_S_4_ inclusions, and small intensive band at 490 cm^−1^ associated with S–S bonds are detectable in the Raman spectra of As_40_S_60_ glass. In contrast with the Raman spectra of stoichiometric glass, the broad band in the region of As–S valence vibrations (~300–400 cm^−1^) in the Raman spectra of corresponding As_40_S_60_ films clearly show the double-peak structure. The simultaneous increases in intensities of 360 cm^−1^ Raman band and bands in the region of molecular and As–As valence band vibrations (100–300 cm^−1^) can indicate the increasing of the concentration of cage-like As_4_S_4_ molecules in As_40_S_60_ films in comparison with those found in the structure of corresponding target glass. As can be seen from intensities of 490 cm^−1^ bands (Fig. 4, curve 1), the concentration of homopolar S–S bonds in the structure of As_40_S_60_ films is larger than in As_40_S_60_ glass. At the same time, the new band at ~270 cm^−1^ is detected in the Raman spectra of As_40_S_60_ films. This band is assigned to the vibrations in As-rich As_4_S_3_ cage-like molecules [28].

The Raman spectra of As_45_S_55_ and As_50_S_50_ glasses are very similar (Fig. 4, curves 2 and 3). The main contributions in the Raman spectra of both glasses originate from As_4_S_4_ cage-like molecules. Weak band at 270 cm^−1^ characteristic of As_4_S_3_ molecules was detected, and no S–S bonds (490 cm^−1^ Raman mode) were found for both glass compositions. The difference in the Raman spectra of As_45_S_55_ and As_50_S_50_ glasses is connected with redistribution of 340 and 360 cm^−1^ band intensities only. In contrast with the glasses, the Raman spectra of As_45_S_55_ and As_50_S_50_ thin films are different. The main differences are connected with the intensity of Raman band at 270 and 360 cm^−1^. These bands are more intensive in the Raman spectra of As_45_S_55_ films indicating the drastic separations of cage-like As_4_S_3_ and As_4_S_4_ molecules from pyramidal network. Also, the very weak band at ~490 cm^−1^ (S–S bonds) is detected in the Raman spectra of both As_45_S_55_ and As_50_S_50_ films.

## Discussion

### Atomic Stoichiometry of As–S films

From the core level spectra of As_*x*_S_100-*x*_ (*x* = 40, 45, 50) thin film surface nanolayers which are shown in Fig. 3, the atomic concentrations and As to S ratios of as-deposited and illuminated by green laser light samples were calculated. The appropriate values are given in Table 2.

**Table 2:** Atomic concentrations, As/S ratio of As_40_S_60_, As_45_S_55_, and As_50_S_50_ nanolayers calculated from XPS data (the values of As/S ratio for the bulk glasses are given in parentheses for comparison) and contribution (area, ±5%) to the core level of each doublet of individual components determined from curve fitting of S 2p and As 3d XPS spectra

Element/Core level/Component	As_40_S_60_		As_45_S_55_		As_50_S_50_	
	As-deposited	Illuminated	As-deposited	Illuminated	As-deposited	Illuminated
As, %	42.9	43.0	48.9	51.0	45.4	45.8
S, %	57.1	57.0	51.1	48.9	54.6	54.2
As/S	0.75 (0.67)	0.75	0.96 (0.82)	1.04	0.83 (1)	0.84
**S 2p:**						
S–As_2_, %	84.8	90.4	93.0	94.3	86.4	89.2
S–SAs, %	15.2	9.6	7.0	5.7	13.6	9.8
**As 3d:**						
As–S_3_, %	90.2	92.1	44.5	16.5	57.5	75.6
As–S_2_As, %	5.7	4.1	43,8	45.8	35.6	21.4
As–SAs_2_, %	4.1	3.8	6.6	27.6	6.9	3.0
As–As_3_, %	–	–	5.1	10.1	–	–

As it can be seen, the thermal evaporation of the bulk chalcogenide glass of As_40_S_60_ composition causes the As_42.9_S_57.1_ composition of deposited thin film. Laser light illumination with near-bandgap photon energy leads to further slight arsenic enrichment. More As-rich thin film in comparisons with target composition is obtained when the As_45_S_55_ glass is evaporated (see Table 2). Further arsenic content increment from 48.9% in the as-deposited sample to 51.0% in the sample illuminated by a green laser light during 90 min takes place. Correspondingly, the appropriate As to S ratio is changed from 0.96 to 1.04. Unexpectedly, the thermal evaporation of As_50_S_50_ glass leads to deposition of thin film with the As/S ratio which is less than for the bulk glass (As_45.4_S_54.6_ composition) (Table 2). The laser treatment of this sample causes small arsenic enrichment, but the ratio between As and S remains far from the appropriate value in the bulk glass.

Such deviations of the thin film stoichiometry from the bulk glasses and further changes to them under the external (laser) influence with photon energy close to the band gap of investigated materials can be understood and explained from a detailed component analysis [29, 30].

### Component Analysis of As_*x*_S_100-*x*_ (*x* = 40, 45, 50) Thin Films Under External Influence

As mentioned above, the fit of core level spectra of all films (before and after treatment) demonstrates the presence of structural units with homopolar bonds (see Fig. 2). Because of sulfur is twofold coordinated and arsenic is threefold coordinated in As-S system, the As_40_S_60_ films in ideal composition should demonstrate the water-like S–As_2_ and pyramidal As–S_3_ components only in their S 2p and As 3d core level spectra, respectively. However, the homopolar As–As bonds were detected in the As 3d core level spectra of all As_*x*_S_100-*x*_ (*x* = 40, 45, 50) nanolayers which is expected from As-enrichment (*x* > 40 at. % As) of their top surface. In accordance with this for the As_40_S_60_ composition, it was found of 5.7% s.u. which are assigned to arsenic bonded to two sulfur and one arsenic atoms, and of 4.1% s.u. which mean the presence of the arsenic bonded to one sulfur and two arsenic atoms (peaks 2, 2’ and 3, 3’, respectively). The contributions of these two As-rich components are much significant in As 3d core level spectra of As_45_S_55_ film surface (see Table 2). Moreover, the fourth component, As–As_3_ is appeared with 5.1% contribution, which is reasonable for calculated composition (As_51.1_S_48.9_). Finally, the thin film obtained by thermal evaporation of the As_50_S_50_ target glass contains 35.6% of As–S_2_As s.u. and 6.9% of As–SAs_2_ s.u. apart from the pyramidal one.

Despite to arsenic enrichment of all three as-deposited As-S samples in comparison with the stoichiometric composition, the S 2p core level spectra contain a component with the homopolar S–S bond (Fig. 2). There is a strong correlation between the As to S ratio of as-deposited samples and the percentage of S–SAs s.u. in the appropriate S 2p core levels. However, the further explanation of the existence of S–S bonds in As-rich structures is needed.

The properties and micro structure of vapor-deposited films depend on the deposition methods and conditions. Therefore, the resulting film structure can be different from the structure of the corresponding bulk glasses as established earlier [31]. The As–As bond formation in the as-deposited As_2_S_3_ film was detected with using X-ray diffraction technique. On the basis of the arsenic enrichment of the film and detected by mass spectroscopy fragmentation into S_2_ and As_4_S_4_ during the evaporation of the bulk As_2_S_3_ glass, the formation of a sheet-like open structure of the film is supposed [31]. Also, it is pointed out that the As–As bonds may be incorporated as S_2_As–AsS_2_ units, as in As_4_S_4_ molecules as determined using extended X-ray absorption edge fine structure and Raman and IR spectroscopy. Apart from this, the dominance of As_4_S_4_ and sulfur molecules in as-deposited films were shown by neutron diffraction study [31]. The mass spectrometry study shows the presence of S_2_ and different AsS particles in the gas phase of As–S system [32]. Therefore, the presence of S–S s.u. (S 2p spectra) in the structure of all As–S films and the appearance of As–S_2_As s.u. in the As 3d core level spectra of even stoichiometric As_40_S_60_ composition can be understood. Moreover, apart from the composition of the target glass, the type of molecules in vapor plays a significant role in the formation of the film composition and structure. This way, the differences in compositions of the films and corresponding source materials can be explained. Additional support of mentioned As-enrichments of top surface As–S nanolayers can be confirmed by Raman spectroscopy. In particular, the biggest arsenic enrichment is found for As–S film deposited from As_45_S_55_ glass where the most significant contribution of As-rich As_4_S_3_ molecules is detected (Fig. 4, curve 2).

Near-bandgap laser light illumination of As_*x*_S_100-*x*_ (*x* = 40, 45, 50) samples causes decreasing of the contribution of components with the homopolar S–S bonds in all the samples (Table 2). This phenomenon was observed in our previous investigations [19] and was explained by the processes of the structural ordering under the laser light illumination. In addition, the decreasing of components with homopolar As–As bonds in the structure of As_40_S_60_ and As_50_S_50_ nanolayers under the near-bandgap laser light illumination was observed (Fig. 2, Table 2). This is in accordance with the results of our in situ under-bandgap laser light illumination of As_2_S_3_ nanolayers [19]. The decreasing of concentration of homopolar As–As bonds in the structure of As_40_S_60_ and As_50_S_50_ films under laser illumination correlates well with the partial disappearance of the S–S bonds and creation of new As–S bonds.

However, the different behavior in compositional and structural changes under laser illumination was observed for As_45_S_55_ films. In contrast with As_40_S_60_ and As_50_S_50_ nanolayers, the increasing of concentration of components with As–As bonds was detected in As_45_S_55_ films as a result of the near-bandgap laser light illumination (Fig. 2, Table 2). Similar increasing was also detected in As_2_S_3_ nanolayers when over-bandgap laser light illumination was applied [19]. This effect was explained by atomic movement of As from deeper to top layers under the laser treatment, leading to As-enrichment of the sample surface. Such movement appears due to a creation of electric field gradient which is driving force on dipoles and charged defects resulting in mass transport.

The larger magnitude of laser-induced changes in As–S system was found for As-rich compositions with As_4_S_4_ inclusions [7]. It should be noted here that XPS spectroscopy reveals the most As-enriched composition of As–S film prepared from As_45_S_55_ glass among studied As_*x*_S_100-*x*_ (*x* = 40, 45, 50) films. Moreover, the significant As enrichment of the As_45_S_55_ sample was confirmed by Raman spectroscopy (Fig. 4, curve 2) where the 270 cm^−1^ Raman band characteristic of As_4_S_3_ molecules show maximal intensity. In this manner, the specific behavior and the structural rearrangement of the As_45_S_55_ nanolayers are conditioned by a considerable number of As-rich s.u., particularly As_4_S_3_. This can stimulate laser-induced mass transport effect resulting in further arsenic enrichment of the sample surface.

### Valence Band Spectra of As–S films

In general, the valence band can be determined as the highest range of electron energies which can be occupied at absolute zero temperature [33]. According to Mott and Devis model, the valence band of amorphous materials contains the states formed by defect centers [34]. For the As–S system, the top of the VB is formed by lone-pair 3p electrons of sulfur (at ~3 eV), as 4p and S 3p levels (bonding electrons) are situated at ~5 and ~7 eV, respectively. Next energy band is located lower than 10 eV and formed by the S 3s and As 4s electrons. These data were confirmed by DFT electronic structure calculations of As- and S-centered s.u. [19].

A qualitative comparison of the VB spectra of As_2_S_3_ nanolayers investigated in situ [19] with the VB spectra of As_*x*_S_100-*x*_ (*x* = 40, 45, 50) films show the differences connected with the presence of additional states at energies ranged from −1.7 to 0.6 eV (Fig. 3). According to the calculated data, the formation of homopolar As–As bonds leads to the appearance of the energy levels in the band gap of the As–S structures [35, 36]. The concentration of As–As bonds in As_*x*_S_100-*x*_ (*x* = 40, 45, 50) films was found to increase in order: As_40_S_60_, As_50_S_50_, and As_45_S_55_ (Table 2). Taking into account, the changes of the concentrations of s.u. with homopolar As–As bonds induced by laser treatment (decreasing for As_40_S_60_, As_50_S_50_ composition and increasing for As_45_S_55_ structure) (Table 2) and intensities of electronic states in the VB spectra of As–S films (from −1.7 to 0.6 eV) (Fig. 3) can be assumed that they are formed by structural units with As–As bonds.

The main changes in the electronic structure of As–S samples induced by laser light illumination can be selected (highlighted regions in Fig. 3). The changes in these regions (denoted as A, B, and C) can clearly be seen from the differential valence band spectra (Fig. 3. bottom). The right highlighted region (A) in the differential VB spectra of As_*x*_S_100-*x*_ (*x* = 40, 45, 50) films points out to the changes of the states in the band gap of the structures. Middle highlighted region (B) indicates that the band gap decreases in the As–S samples of the As_40_S_60_ and As_50_S_50_ compositions and increases in the As_45_S_55_ structure under the near-bandgap laser light illumination. It should be noted that these results correlate with the shift of absorption edge measurements. And finally, left marked region (C) demonstrates common decreasing of the concentration of S–S homopolar bonds in all As–S films under laser treatment (see Figs. 2 and 3, Table 2).

### Profilometry Analysis of Laser Induced Relief Formation in As_*x*_S_100-*x*_ (*x* = 40, 45, 50) Films

**Fig. 5:**
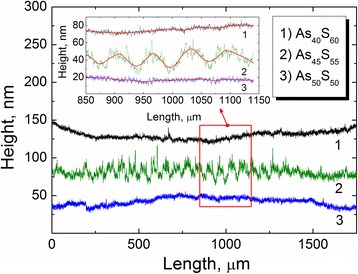
Profiles of the surface of As_40_S_60_, As_45_S_55_, and As_50_S_50_ thin films illuminated by green (λ = 532 nm) laser light through the copper grid

As it was mentioned above, the increasing of the components with the As–As bonds under the near-bandgap laser light illumination takes place due to a creation of electric field gradient resulting in mass transport. In order to examine of this effect, the additional experiments were performed. The as-deposited films of all three compositions were illuminated by green laser light through the copper mash with grating period of 60 μm during time sufficient for saturation of shift of absorption edge, measured previously (see previous chapter). Then, the surface of irradiated As–S samples was examined by AMBIOS XP-1 type profilometer with 10 nm vertical resolution (stylus tip radius—2.5 μm). For the profilometer measurements, a 0.5 mg load was applied. This load was small enough to make the accurate profiling without destroying the surface morphology. Results are shown in Fig. 5.

As can be seen, the laser light illumination does not change the shapes of the surfaces of both As_40_S_60_ and As_50_S_50_ thin films. Thus, it can be concluded that laser illumination was not influenced on morphology of the surface of these samples. However, the different behavior in laser-induced transformations and surface morphology changes was discovered for As_45_S_55_ thin film (see Fig. 5, curve 2). It is clearly seen that under the laser light illumination the “wave-like” relief on the surface of As_45_S_55_ film is formed. The parameters of the induced grating can be seen in the insert of Fig. 5. This relief corresponds to the period of the mash grating.

Taking into account that the As_45_S_55_ composition demonstrates peculiarities and opposite induced phenomena (shift of the absorption edge, stoichiometry and local structure changes, untypical VB shift, and shape changes) in comparison with the As_40_S_60_ and As_50_S_50_ thin films and contains the largest concentration of As_4_S_3_ cage-like molecules, it can be concluded that the presence of polar As_4_S_3_ molecules (which are sensitive to the electric field generated by the laser) is responsible for observed laser-induced mass transport effect. The drift and re-arrangements of this molecules results in photoexpansion of illuminated areas. The observed phenomena can be used for optical grating formation, controlled laser surface modification, laser induced surface activation, etc.

## Conclusions

The local and molecular structures of As_*x*_S_100-*x*_ (*x* = 40, 45, 50) thin film surface and their transformations induced by coherent near-bandgap laser illumination have been investigated using XPS and Raman spectroscopy. The optical properties and induced transformation of surface morphology of As–S nanolayers have been also studied by means of absorption edge spectroscopy and 2D profilometry.

A significant difference in surface stoichiometry between amorphous As–S films and composition of corresponding target glasses was established, and it was found to be related with the peculiarities in molecular constituent of gas phase during the deposition process, indicating that the type of molecules in vapor plays a crucial role in resulting film composition. Near-bandgap laser illumination decreases the concentration of the homopolar S–S bonds in the structure of all As_*x*_S_100-*x*_ (*x* = 40, 45, 50) nanolayers. However, the decreasing of the concentration of homopolar As–As bonds upon laser illumination was observed in the structure of As_40_S_60_ and As_50_S_50_ films only. In contrast with As_40_S_60_ and As_50_S_50_ films, the contribution of As–S_2_As and As–SAs_2_ components and appearance of a new As-rich As–As_3_ s.u. in the structure of As_45_S_55_ thin film during laser illumination were detected. Moreover, this particular film (As_45_S_55_) demonstrates peculiarities in laser-induced shift of the absorption edge, in Raman spectra, and finally, in effect of induced surface morphology transformation.

The results of Raman investigation of As–S films indicate the presence of As-rich As_4_S_3_ molecules in the structure of As_45_S_55_ nanolayers in largest concentration among studied samples. Therefore, the As_4_S_3_ molecules were found to be responsible for drastic difference in behavior of absorption edge spectra and surface morphology transformation of As_45_S_55_ nanolayers during near-bandgap laser illumination. The presence of these As_4_S_3_ structures in the structure of As_45_S_55_ nanolayers results in laser-induced mass transport effect observed for this material and can be useful for optical grating formation and related external nanofabrication technologies.

## Abbreviations

BE: Binding energy
ChG: Chalcogenide glass
FWHM: Full width at half maximum
VB: Valence bands
XPS: X-ray photoelectron spectroscopy
